# Reduction in Healthcare Resource Utilization Following Treatment With a Home-Based Footworn Device in Patients With Knee Osteoarthritis: A Retrospective Claims Analysis

**DOI:** 10.36469/001c.117155

**Published:** 2024-05-16

**Authors:** Josh Mark, Shirley Shema-Shiratzky, Joel Sommer, Tim Nolan, Ganit Segal

**Affiliations:** 1 AMA Research Group, Tel-Aviv, Israel

**Keywords:** knee osteoarthritis, noninvasive intervention, healthcare resource utilization

## Abstract

**Background:** One in 7 US adults has knee osteoarthritis (OA) and almost two-thirds of them suffer from low back pain. OA is the third most rapidly rising condition associated with disability and leads to a significant burden on the healthcare system and society. **Objective:** This study looked at the healthcare resource utilization (HCRU) in patients with knee OA and low back pain before and after the utilization of a new, home-based, noninvasive, biomechanical intervention. **Methods:** This was a retrospective claims analysis of 585 patients treated with a personalized, noninvasive, home-based, biomechanical treatment that aims to alleviate knee pain and improve function (AposHealth®). The date of the first AposHealth claim was the index date. Data prior to the index date and post-index date were used to monitor changes in HCRU while in treatment. Descriptive statistics, including frequencies, means and standard deviations, were used to present patient characteristics. To standardize the results, an average monthly claims data rate was calculated and an expected annual rate was extrapolated. Annual HCRU rate per 1000 members was calculated. **Results:** HCRU decreased after utilizing the new intervention including a decrease of 79% in diagnostic claims, a 70% decrease in outpatient services, a 22% decrease in non-operative treatments, a 61% decrease in pain medications including an 85% drop in opioids use, and a 44% decrease in intra-articular injections. The pre-index estimated rate for total knee replacement (TKR), which is based on existing literature, was 15.1%, whereas the post-index rate of TKR was 0.9%. **Conclusions:** Patients with knee OA treated with a home-based, noninvasive, biomechanical intervention incurred fewer healthcare resources, leading to an overall reduction in the cost of care.

## INTRODUCTION

Knee osteoarthritis (OA) is a prevalent degenerative joint disease characterized by the progressive breakdown of articular cartilage, leading to pain, stiffness, and impaired mobility.^1,2^ It is a leading cause of disability and a significant burden on healthcare systems worldwide.^1,3^ One in 7 US adults (32.5 million people) has knee OA,^2^ and almost two-thirds of them suffer from low back pain.^4^ The prevalence of OA is the third most rapidly rising condition associated with disability, just behind diabetes and dementia,^5^ and the numbers are expected to grow with the aging of the population. It is estimated that by 2032, the number of people with knee OA will increase, and 1 in 6 people older than 45 years will seek physician consultation due to knee pain.^6^ In the United States, the estimated lifetime cost of a patient diagnosed with knee OA is $140 300; over half of all patients will have a total knee replacement (TKR).^7^ The overall economic burden of knee OA is estimated at $27 billion annually^7^ and is expected to rise with the increase in prevalence, highlighting the need for better disease management, new nonsurgical interventions, and technologies that will be effective and ideally demonstrate cost savings.

The management of knee OA involves a multidisciplinary approach, including nonpharmacological, pharmacological, and surgical interventions.^8^ Nonpharmacological interventions may include weight management, exercise programs, physical therapy, assistive devices, and patient education. Pharmacological treatments often involve analgesics, nonsteroidal anti-inflammatory drugs (NSAIDs), intra-articular injections, and, in severe cases, opioids. Additionally, when conservative treatments fail, surgical interventions such as TKR may be necessary.^8^ Several factors, including patient demographics, disease severity, comorbidities, geographical location, socioeconomic status, and healthcare system characteristics influence healthcare resource utilization (HCRU) in knee OA, such as diagnostic examinations, pharmacological interventions, and noninvasive and invasive interventions. There is no clear care pathway, and the current literature suggests conflicting evidence regarding the clinical effect of the recommended interventions for knee OA.^9^ For example, Roos and Nigel suggest that knee OA is amenable to early prevention and treatment, primarily by focusing on neuromuscular and proprioceptive training and improving muscle function.^10^ Alternatively, recent evidence suggests conditional recommendation of intra-articular injections with only short-term effect and potential accelerated cartilage damage.^11,12^ Optimizing and personalizing HCRU, as well as introducing new interventions and presenting high-quality research on current interventions, can help reduce the burden of knee OA on healthcare system and society.

The purpose of the current study was to report on HCRU for patients with knee OA and low back pain before and after the utilization of a new, home-based, noninvasive, biomechanical intervention (AposHealth®) that was found safe and clinically effective.^13–15^ The treatment aims to reduce pain and retrain gait using a biomechanical footworn device. To the best of our knowledge, there is no information about the effect of this intervention on the utilization of nonsurgical healthcare resources and associated costs in the United States. We hypothesized that utilizing this intervention would reduce the overall HCRU for patients with knee OA.

## METHODS

The study was a retrospective claims analysis of a cohort of members covered by a state payor who received treatment for knee and back pain as part of a pilot program that aims to utilize a new noninvasive, home-based, biomechanical intervention. Diagnostic criteria were used to determine patients’ eligibility for the program. The intervention may be considered medically necessary when there is a diagnosis of moderate or advanced knee joint disease, supported by a clinical diagnosis of knee OA, or complaints of chronic low back pain. A complete description of the inclusion criteria is summarized in **Table 1**. All data for this study were drawn from a healthcare claims dataset while keeping patient records de-identified. Variables in the dataset include patient characteristics, diagnoses, procedures, medications, and payment amounts. Patients received treatment between May 2021 and June 2022 from 13 physical therapy (PT) providers across New York City. All PT providers were trained to administer the intervention. Potential patients were identified in the clinics. If patients had a diagnosis of a primary knee or back condition and met eligibility, the PT would start a clinical evaluation and device set up following a specific methodology, as detailed below. Once the calibration of the device was completed and patient started treatment, the PT would submit a claim with the payor.

**Table 1.** Eligibility Criteria of the Home-Based, Noninvasive Biomechanical Intervention

Medically Necessary	Not Medically Necessary
A diagnosis of moderate or advanced knee joint disease or low back pain supported by a clinical diagnosis including the following diagnostic codes: M170, M1710, M1711, M1712, M179, M25561, M25562, M25569, M47817, M5136, M5186, M5416, M5417, M5430, M5431, M5432 M5441, M5451, M549, and corresponding to the following indications: Bilateral primary osteoarthritis of knee; unilateral primary osteoarthritis, unspecified knee; unilateral primary osteoarthritis, right knee; unilateral primary osteoarthritis, left knee; osteoarthritis of knee, unspecified; pain in right knee; pain in left knee; pain in unspecified knee; spondylosis without myelopathy or radiculopathy, lumbosacral region; other intervertebral disc degeneration, lumbar region; other intervertebral disc disorders, lumbar region; radiculopathy, lumbar region; radiculopathy, lumbosacral region; sciatica, unspecified side; sciatica, right side; sciatica, left side; lumbago with sciatica, right side; lumbago with sciatica, left side; vertebrogenic low back pain; dorsalgia, unspecified. Documentation of persistent pain that is not controlled despite optimal, conservative pain management: Description of the pain (onset, character, aggravating, duration, and relief factors), analgesics and the treatment modalities used. Documentation of functional limitations that interfere with ADLs: Specific limitation of ADLs; Evaluation of safety issues, eg, fall risk Documentation of a history of conservative medical therapy that has been tried and failed including but not limited to ≥1 of the following: Activity modification Structured land-based programs including strengthening and/or cardio and/or balance training/neuromuscular exercise Mind-body exercise including tai chi or yoga Physical therapy that includes flexibility and muscle strengthening exercises NSAID Therapeutic intra-articular injections as appropriate Weight loss efforts as appropriate	The interventions will be considered contraindicated and not medically necessary with the presence of the following: Active infection of the knee joint or back or an active infectious process anywhere in the body (eg, systemic bacteremia); or Patient requires a cane or walker both indoors and outdoors History of ≥2 unexplained falls within the last 12 mo Severe neurological, psychiatric, or comprehension issues preventing an understanding of how to use the device Severe balance or vertigo issues

All patients signed a consent acknowledging that their data might be used anonymously for research purposes. Patients received a de-identified code that was used during the analysis and ensured anonymized data.

### Outcomes and Data Collection

**Figure 1.**
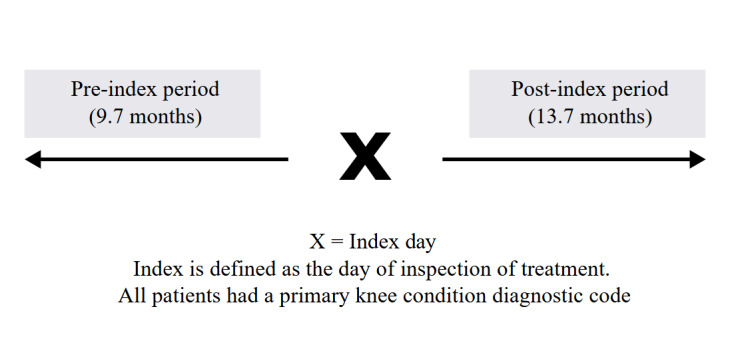
Pre-Post Index Period

Patients in the current study served as their own control. The date of the first AposHealth claim was the index date and reflects a point in time where data was analyzed pre- and post-intervention. More specifically, the date of device calibration and treatment initiation is the index for cost and utilization comparison. Incurred dates from May 1, 2020, to the earliest activity date of T1999 minus 1 day comprise the pre-treatment time frame, while incurred dates from the earliest activity date of T1999 to June 30, 2022, comprise the post-treatment time frame. Data prior to the index date (pre-index) was used to describe HCRU and associated costs before commencing treatment. It is postulated that pre-index HCRU represents the current incur of healthcare services to treat patient’s knee and/or back condition. Data after the index date (post-index) was used to monitor changes in HCRU while in treatment. It is postulated that changes in HCRU might be attributed to the intervention; however, the design of this study does not support cause and effect and in practice will present HCRU trends. The average pre- and post-index time was 9.7 months and 13.7 months, respectively (**Figure 1**).

Pre- and post-index HCRU and costs were exported by the payor, using a claim code that is associated with the intervention, and shared via secured and encrypted methods using de-identified numbers with no protected health information. Direct HCRU and costs were defined by the subset of claims with diagnoses or procedures for knee OA and low back pain and were classified into the following subgroups:

1. Knee diagnostics including x-ray, ultrasound, computed tomography, and magnetic resonance imaging
2. Knee outpatient services (OP) including ≥1 ER visit, ≥1 OP office visit (orthopedics), ≥1 other OP office visit (nonorthopedic claim–related). Of note, ≥1 OP office visit represents a first time visit to a doctor, while ≥1 other OP office visit reflects a visit from an established patient
3. Nonoperative knee treatments including knee orthosis, occupational therapy, physical therapy, and other therapy
4. Knee pain medications including ≥1 NSAID prescription, ≥1 opioid prescription, ≥1 other pain medication
5. Intra-articular knee injections including corticosteroid, hyaluronic acid, guidance – general, general – large joint
6. Knee surgery including arthrotomy, arthroscopy, and TKR

### Intervention

**Figure 2.**
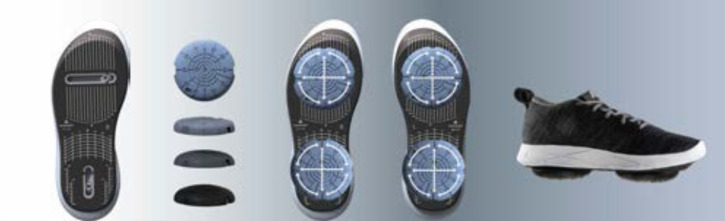
Apos System Sole platform includes 2 mountain rails and a positioning matrix. Biomechanical pods are available in different degrees of convexity and sizes. Biomechanical pods can be configured on the sole platform with flexible positioning for personalized biomechanical placement.

All patients received an FDA-cleared personalized calibrated foot-worn device and a home-based treatment that aims to alleviate knee pain and improve function (AposHealth®) in patients with musculoskeletal conditions. A description of the device and treatment methodology was reported by Shema-Shiratzky et al.^16^ In short, it includes a unique footwear (Apos device; **Figure 2**) and uses 2 convex pods attached to the plantar surface of the sole. A specially trained physiotherapist calibrates (ie, customizes the location and size of the pods) the device to reduce pain in the knee or back while walking. A complete calibration includes a pair of shoe-like device with customized pods at the bottom of the shoe that leads to functional re-alignment and revised load distribution of the kinematic chain and generates neuromuscular stimulation to retrain neuromuscular control. Calibration is determined based on patients’ symptoms, physical examination, and computerized gait assessment. The aim is to link between patient’s symptoms, functional limitation and gait abnormalities and compensation mechanisms. The adjustment of pod location manipulates the foot’s center of pressure, shifts the ground reaction force vector, and reduces pressure on the joint immediately.^17,18^ The convexity of the pods induces controlled perturbation by decreasing the foot base of support, therefore creating a challenge to gait and posture stability. This controlled instability triggers neuromuscular training response and alters patients’ gait patterns.^19,20^ After the initial calibration, patients are instructed to wear the device daily for short periods of time at home or work while performing task-specific activities (ie, daily living). Patients are instructed to gradually increase wear time from approximately 20 minutes per day initially, reaching up to 2 to 3 hours per day. This home-based training allows patients to manage their symptoms and integrate the training into their daily routine, further inducing a carry-over beneficial effect to usual walking (without the device) over time.^14^ Patients were requested to return to follow-up appointments in which reassessment of clinical outcomes and gait patterns was performed and the calibration of the device and treatment plan were adjusted as needed. Typically, patients are advised to return to 2 to 3 follow-up appointments, with the first after 4 weeks of treatment and subsequent follow-ups 2 to 3 months apart.

### Statistical Analysis

Descriptive statistics, including frequencies, means, and SD, were used to present patient characteristics. A count of HCRU claims was performed. To standardize the results, an average monthly claims data rate was calculated, and an expected annual rate was extrapolated. Annual HCRU rates per 1000 members were calculated. This study examined a cohort of patients pre- and post-intervention. The claims review did not include the rates of TKR pre-index since in the current study design (ie, pre-post index) having a TKR claim means there will not be another one. Including those with a TKR claim will bias the results of the study (in favor of the intervention) and therefore the expected TKR rates in this population based on existing literature were used. Moreover, the pre-index TKR utilization rate might not reflect accurate rates due to selection bias (patients in the current analysis do not represent the general knee OA population). Therefore, we used data from the Milliman report,^21^ which looked at TKR rates in patients with knee OA, split into mild knee OA (ie, those receiving core interventions) and moderate-severe knee OA (ie, second line of care), to estimate the pre-index TKR rates.

The payor’s resources costs were used to calculate the implied spending. All spending amounts were taken from the time in which they were incurred (April 2019–April 2022) and were not adjusted to reflect inflation. Cost differences were measured by comparing utilization before treatment to utilization after treatment and multiplying the difference in utilization rate by the average cost for the service/procedure for the entire study period. A comparison between the pre-and post-index spending reflects the differences in spending pre- and post-index.

## RESULTS

A total of 585 patients, 430 with primary knee condition and 155 with primary back pain, were identified and included in the study. The average (SD) age was 53.8 (10.9) years, 76% were females.

The HCRU decreased after the utilization of the new intervention, as summarized in **Table 2.** In conjunction, the post-index HCRU costs decreased by 90% from $8 557 046 pre-index to $867 877 post-index. When accounting for the cost of care of the intervention, the overall cost difference was $3 689 169 annually per 1000 members, corresponding with a 43% decrease in overall costs and a $3689 savings per patient (**Table 3**). The main driver for cost reduction was differences in knee surgery, primarily TKR followed by cost reduction associated with outpatient services.

**Table 2.** Healthcare Resource Utilization Rates Pre-index and Post-index

	Pre-index Average Utilization/1000	Post-index Average Utilization/1000
Knee surgery		
Total knee replacement	151.00	9.00
Arthroscopy	48.51	0.00
Arthrotomy	0.00	0.00
Knee diagnostics		
Computed tomography (CT)	10.55	7.50
MRI	122.34	25.50
Ultrasound	27.42	4.50
X-ray	797.33	165.02
Knee injections		
Corticosteroid–general	885.92	225.03
Guidance general	52.73	31.50
Hyaluronic acid	742.49	801.10
Injection–general large joint	521.01	175.52
Knee nonoperative treatment		
Knee orthosis	21.09	4.50
Occupational therapy	0.00	1.50
Other therapy	6277.38	5052.63
Physical therapy	326.95	123.02
Knee OP		
≥1 ED visit	0.00	3.00
≥1 OP office visit	284.76	63.01
≥1 other OP office visit	1023.03	324.04
Knee pain medications		
>1 NSAID prescription	21.09	10.50
>1 opioid prescription DX	21.09	3.00
>1 other pain medication	0.00	3.00

An average monthly claims data rate was calculated, and an expected annual rate was extrapolated. Annual HCRU rates per 1000 members were calculated. For outpatient services, ≥1 OP office visit represents a first time visit to a doctor, while ≥ 1 other OP office visit reflects a visit from an established patient.

Abbreviations: DX, diagnosis; ED, emergency department; HCRU, healthcare resource utilization; MRI, magnetic resonance imaging; NSAID, nonsteroidal anti-inflammatory drugs; OP, outpatient.

**Table 3.** Healthcare Resource Utilization Costs Pre-index and Post-index

**Metric**	**Utilization Pre-index** **(No. of claims)**	**Utilization Post-index** **(No. of claims)**	**Reduction (%)**	**Pre-and Post-index Differences ($)**
Knee surgery	199.51	9.00	-95	7 374 018
Knee OP services	1307.79	390.05	-70	125 545
Knee diagnostics	957.64	202.53	-79	85 804
Knee nonoperative treatment	6625.42	5181.65	-22	56 952
Knee injections	2202.14	1233.15	-44	46 338
Knee pain medications	42.19	16.50	-61	512
Total cost difference per 1000 users				7 689 168
Total cost difference per 1000 users, accounting for cost of care				3 689 168

Knee surgery including arthrotomy, arthroscopy, and total knee replacement; knee outpatient services including ≥1 emergency department visit, ≥1 outpatient office visit (orthopedics), ≥1 other outpatient office visit (non-orthopedic claim–related); Knee diagnostics including x-ray, ultrasound, computed tomography, and MRI; nonoperative knee treatments including knee orthosis, occupational therapy, physical therapy, and other therapy; intra-articular knee injections including corticosteroid, hyaluronic acid, guidance – general, general – large joint; knee pain medications including ≥1 NSAID prescription, ≥1 opioid prescription, ≥1 other pain medication.

Abbreviations: NSAID, nonsteroidal anti-inflammatory drugs; OP, outpatient.

The proportion of patients with a diagnostic claim decreased by 79%. There were 958 diagnostic claims pre-index compared with 203 post-index. The proportion of OP services claims decreased by 70%. There were 1308 OP service claims (indicating ≥1 claim per patient) compared with 390 post-index. The proportion of patients with a nonoperative treatment claim decreased by 22%. There were 6626 nonoperative treatment claims pre-index compared with 5182 post-index. The proportion of pain medication claims decreased by 61%. There were 43 claims pre-index compared with 17 claims post-index. More specifically, there were 21 claims for at least 1 opioid prescription pre-index and 3 claims post-index, a reduction of 86%. There were 21 claims for at least 1 NSAID prescription pre-index and 11 claims post-index, a reduction of 48%. The proportion of intraarticular injections had decreased by 44%. There were 2203 claims of injections pre-index compared with 1234 post-index. The proportion of knee joint surgery decreased by 93%. The estimated TKR rate, based on existing literature, was 15.1%, whereas the post-index rate of TKR was 0.9%.

## DISCUSSION

The current study reports the HCRU and implied costs in patients with knee OA treated with a new noninvasive intervention. By better understanding HCRU and costs, healthcare providers, policymakers, and researchers can develop targeted strategies to improve patient outcomes, enhance resource allocation, and mitigate the economic burden of knee OA. The results of the current study suggest that with the utilization of this intervention, overall HCRU may decrease. Consequently, the overall direct cost of care may decrease as well. The results correspond with a previous study that reported HCRU changes after utilizing this intervention as measured with a self-reported patient survey. Those results suggested that 44% of patients discontinued consuming pain relief medications, 46% stopped receiving PT, and 64% ceased intra-articular injection.^16^ The current study suggests a reduction in the rates of TKR (of 93%), diagnostic claims (79%) and prescribed pain relief medication (61%). Based on the results of Kurtz et al, in 2030 there will be 3.48 million TKRs annually, a 673% increase from 2005 rates.^22^ Therefore, any intervention that helps reduce the number of procedures will directly affect the economic burden, as most of the direct costs of knee OA are associated with surgery. Specifically, previous studies suggest that patients that were treated with this intervention reported a significant reduction in pain and improvement in function and quality of life^13,23–26^ due to changes in gait biomechanics, specifically, a reduction in knee frontal and sagittal moments^14,27^ and better neuromuscular control and synchronized muscle activation patterns.^20^

With the lack of effective noninvasive therapeutic options for patients with knee OA, clinicians prescribe pain relief medication in the hope of relieving patients’ symptoms, often underestimating their potentially harmful consequences. Opioid overuse and misuse impacts patients globally. In the US, the proportion of elderly OA patients being prescribed opioids at least once had increased from 31% in 2003 to 40% in 2009.^28^ Supposing that the upward trend in the rate of opioid prescriptions over the last decade remains constant, the costs will increase from $25.1 million in 2015 to $72.4 million in 2030,^29^ not accounting for the indirect costs associated with overutilization. Clinically, the benefit of opioid treatment, regardless of preparation or dose, does not outweigh the harm it might cause.^30^ Similarly, NSAID use is often not appropriate for patients with comorbidities or for long-term use.^30,31^ Since pain medications provide only symptomatic relief, it may be assumed that once they are started, patients will be reliant on them for years. It may be assumed that patients in the current study felt symptomatic relief to the extent that pain relief medications were no longer necessary (86% and 48% reduction in opioids and NSAIDs post-index, respectively). This was also evident in a previous study that reported a 58% reduction in pain relief medication while treated with this intervention compared with controls.^32^

Some limitations should be acknowledged. First, this study documented the HCRU of patients pre- and post-utilization of a new intervention. In some cases, using the same cohort to monitor HCRU might bias the results, especially when looking at resources that are typically done periodically (such as diagnostics and some OP resources). Applying a randomized controlled trial or matched-control methodology is challenging since there is no clear care management protocol. Patients are often prescribed with interventions in a shared-decision process based on medical history, characteristics, and preference. The current methodology cannot support a cause and effect that can attribute savings to the intervention rather than more appropriately reflect trends. That said, the program enrolls patients with moderate to severe symptoms who failed core interventions and second-line interventions, placing them at high risk for TKR. In addition, some procedures are routinely utilized (pain medications, intra-articular injections), and a reduction in their utilization probably reflects a genuine decrease in demand. Second, the cohort includes patients with knee and back pain. In addition, some patient demographic information is not available. Although most patients had a primary knee condition (73%), some had a primary back condition. Since we applied a methodology of pre- and post-index claims analysis, we believe this had a minor effect on the results. Third, some costs were not available and accounted for, including inpatient visits and services and indirect costs, specifically ones associated with TKR (work absenteeism). To accommodate for this gap, we used estimates as a proxy to account for both inpatient and outpatient services; however, results should be interpreted carefully. Future studies should be designed to capture the overall direct and indirect costs associated with a disease with and without the utilization of this intervention. Fourth, we cannot confirm the reason for the reduction in HCRU. Based on previous studies, we postulate that the reduction in HCRU is a result of a significant clinical and biomechanical effect. Previous studies suggest that the treatment leads to reduction in pain, improvement in function, quality of life, and gait patterns. This may lead patients to be more active, less reliant on pain relief medications/injections, and have fewer doctor visits associated with their knee and/ or back condition. Fifth, radiographic evidence of knee OA was not available, and we could not determine disease severity. However, the program applies a set of diagnostic criteria to determine eligibility which includes moderate-severe patients, clinically diagnosed with knee OA who failed core interventions. Lastly, the study included patients from New York City, and one should cautiously generalize the results to other populations. Future studies should evaluate HCRU with and without this intervention in other patient populations; however, based on previous studies, it is reasonable to assume results will be similar.

## CONCLUSION

This study suggests that patients with knee OA treated with a home-based, noninvasive, biomechanical intervention incur fewer healthcare resources, leading to an overall reduction in the cost of care primarily by a reduction in TKR and pain relief medication.
